# A comparative study of 22-channel water-perfusion system and solid-state system with 36-sensors in esophageal manometery

**DOI:** 10.1186/1471-230X-12-157

**Published:** 2012-11-07

**Authors:** Kun Wang, Li-ping Duan, Ying Ge, Zhi-wei Xia, Zhi-jie Xu

**Affiliations:** 1 Department of Gastroenterology, Peking University Third Hospital, 49 North Garden Rd., Haidian District, Beijing, 100191, People’s Republic of China

**Keywords:** 22-channel water-perfusion manometry, Solid-state manometry (SSM) with 36 sensors, Pressure measurements, Patients’ tolerance, Operators’ convenience, Comparative study

## Abstract

**Background:**

To compare the characteristics between 22-channel water-perfusion manometry (WPM) and solid-state manometry (SSM) with 36 sensors of the pressure measurements, as well as patients’ discomfort indices in nose and pharynx, the preparation and operation time of the manometry.

**Methods:**

12 volunteers were included in the study. Each of the volunteers underwent esophageal manometry by both 22-channel water-perfusion catheter (WPC) and solid-state catheter (SSC) with 36 sensors in random order, and separated by 30 min. The subjects gave a VAS score soon after each test. Non-parametric tests were used to analyze the differences and Bland-Altman plots were used to assess the consistency of the two systems.

**Results:**

During the wet swallows, there were significant differences between the two systems in three measurements of location of lower esophageal sphincter (LES) upper margin (Z = -2.11, P = 0.035), LES relax ratio (Z = -2.20, P = 0.028) and IRP4s (Z = -2.05, P = 0.041). During the jelly pocket swallows, LES relax ratio measurements of the two systems showed significant differences (Z = -2.805, P = 0.005). Further Bland–Altman plots analysis presented good agreement between the two systems measurements of location of LES upper margin, LES relax ratio and IRP4s. The discomfort indices of subjects’ nasal sensation were higher when inserting the solid-state catheter [5(3.75-5)] than water-perfusion one (2.5(2-4)) (Z = -2.471, P = 0.013), as well as the discomfort indices of pharyngeal sensation (7.5(4.75-9) vs. 4.5(3.75-6.5)), (Z = -2.354, P = 0.019). The preparation time for WPC was 40(39-41) minutes, which was much longer than that for SSC 32.5(31.75-33) minutes, (Z = -3.087, P = 0.002). And the nurses reported it’s much easier to insert WPC (Z = -3.126, P = 0.002).

**Conclusions:**

In conclusion, most pressure measurements were consistent between WPM and SSM. Patients tolerated better with WPC, while for operators, the SSC presented more convenient.

## Background

Esophageal manometry is the standard procedure used to accurately define esophageal motor function via providing measurements of esophageal pressures, peristalsis and coordination. All GI manometry systems consist of two hardware components: a pressure sensor/transducer, which is able to sense changes in intraluminal pressure and convert what is detected into an electrical signal, and a recording device that amplifies the signal and stores it [1]. The introduction of the first pressure measurements of the esophagus was in the late 1950s. High resolution manometry (HRM) is the latest development in recent years and there are two main types of HRM recording systems that depend on assemblies that incorporate intraluminal solid-state transducers, and those that use perfused assemblies connected to external transducers [2]. The water-perfusion manometry (WPM) allows recording of multiple pressure channels from one catheter and is relatively inexpensive. However it has several disadvantages. It has a limited frequency response, is difficult to set up and use, needs skilled personnel for equipment maintenance, and it is prone to artefacts due to movement of the connecting tubing or air bubbles in the system [3]. The solid-state manometry (SSM) is good high frequency response, application in ambulatory subjects and ease of use, but it is very expensive and somewhat fragile and requires cleaning and sterilization after each use. Draganov et al [4] and Florisson et al [3] conducted trials comparing solid-state catheter (SSC) and triple-lumen water-perfusion catheter (WPC) for sphincter of Oddi manometry done at the time of ERCP and for anorectal manometry in pig models, and stated the use of the SSC system carries potential advantages such as the SSC is simpler and easier to set up and use because the equipment has fewer components and is freely mobile and the use of the SSC may decrease the risk of post-ERCP pancreatitis. However, they performed the tests at the time of ERCP or in animal models, offering little information about the patients’ nasal and pharyngeal sensation during esophageal manometry. Besides, the patterns of luminal pressure perception and conversion of the pressure signal into an electrical signal are different, pressure measurements from the two manometers may exist differences. Castell et al [5] and Pursnani et al [6] performed trials comparing SSC and WPC focused on pharyngeal, upper oesophageal sphincter and lower oesophageal sphincter pressure measurement. However, in the times of their trials, the manometry catheter was only equipped with small number of transducers. By now, in our knowledge, though a few reports mentioned the differences of the two systems [7], there are scarcely any reports about the detailed differences of comprehensive motility parameters of esophagus between the two HRM systems. The aim of the current study is to compare the characteristics between 22-channel WPM and SSM with 36 sensors of the pressure measurements, as well, patients’ discomfort indices in nose and pharynx, the preparation and operation time of the manometry.

## Methods

### Ethics

This study was approved by the ethical committee of Peking University Health Science Center, and all subjects gave informed consent in writing before commencement of the study.

### Subjects

Volunteers were included in the studies. The age was 18 ~ 65 years old. They were healthy volunteers or patient volunteers with poststernal discomfort or dysphagia. All subjects underwent electrocardiogram, gastroscopy and esophageal manometry. Criteria for exclusion from the study included such diseases as peptic ulcer, digestive cancer, previous abdominal surgery, diabetes mellitus and severe cardiac-cerebral diseases such as cerebral infarction.

### Equipments

**Figure 1:**
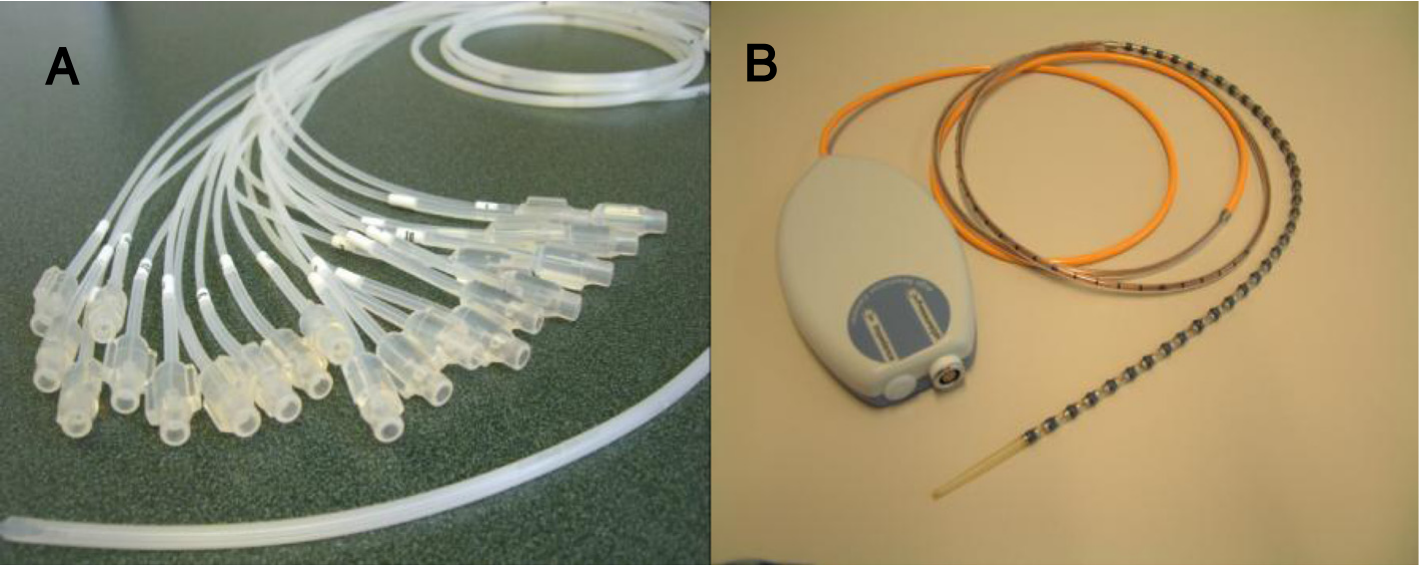
A: The 22-channel water-perfused catheter B: The solid-state catheter with 36 sensors.

The water-perfused high resolution manometry system (Medical Measurement Systems, Enschede, The Netherlands) was equipped with a catheter which is composed of 22 thin polyvinyl tubes (channel P1-P22). The outer-diameter of catheter was 4.2 mm. Each tube has a unidirectional side-hole. The P1 channel was in the distal terminal of the catheter, and 5 cm above it was P2 channel. Between each of the points of P2-P7, 1 cm interval was designed to accurately measure the LES motility function. From P8 to P22, the interval was 2 cm between one to the next Figure 1A presented the WPC. Before inserting, the skilled nurse needed to link the catheter to the pump and exhaust the air bubbles in the catheter [8]. After each use, cleaning and sterilization was required. The Solid-state high resolution manometry system (Medical Measurement Systems, Enschede, The Netherlands) was equipped with a catheter with 36 transducers (Unisensor AG, Attikon, Switzerland) which connects directly to the polygraph recording device. The sensors are unidirectional and covered by the circumferential soft membrane with fluid inside. The luminal pressure acts on the membrane and was shared by the fluid, so the sensors actually perceive the average luminal pressure *in situ*. The outer-diameter of thinnest part of this catheter was 3.8 mm and 5.4 mm of each transducer site. The interval of each transducer point was 1 cm. Figure 1B presented the SSC. After each use, cleaning and sterilization was also required.

### Protocol

Each of the 12 volunteers underwent esophageal manometry by both WPC and SSC in random order, and separated 30 min, according to the standard clinical protocol of the gastrointestinal motility center of Peking University Third Hospital. During the manometry process, the subjects lay, and the catheter, needed exhausting air and standard if with WPC, was passed transnasally into the stomach. After recording the LES and UES rest pressure and length, esophageal body motility function analysis was performed with the point located 4 cm above the LES and 4 cm below the UES using a series of 10 wet swallows with 5 ml water and 10 jelly pockets swallows with 2-cm diameter, each separated by 30 sec. For each esophageal procedure, variables captured included the proximal LES and UES location, LES and UES length, rest pressure, relax ratio, the proximal and distal esophageal body contraction intensity and duration. And the pressure topography parameters previously established by the Chicago Classification were also compared between the two systems. The contractile front velocity (CFV) was defined as the slope of the line connecting the points on the 30 mmHg isobaric contour at the proximal and the distal margin of the distal esophageal segment. Distal contractile integral (DCI) quantifies product of amplitude × duration × length (mmHg-s-cm) of the distal esophageal contraction > 20 mmHg from proximal to distal pressure troughs. Integrated relaxation pressure (IRP) represents Mean EGJ pressure measured with an electronic equivalent of a sleeve sensor for four contiguous or non-contiguous seconds of relaxation in the ten-second window following deglutitive UES relaxation. [9]. Subjects gave Visual Analogue Scale (VAS) scores (0-10 cm) about the nasal and pharynx discomfort soon after each manometry procedure. The VAS scales were 100 mm vertical lines anchored with “no discomfort” at the bottom and “worst imaginable pain” at the top. And the skilled nurse also filled out a questionnaire about the catheter preparation time, cleaning and sterilization time, motility process time which recorded by a timer and the VAS difficulty scores of catheter insertion.

### Statistic analysis

Data are shown as median (Q). Statistical comparisons between the groups (SSC vs. WPC) were made using Wilcoxon two-related-samples test. A p value < 0.05 was considered significant. Individual pressure measurements were plotted using the SSC and WPC, respectively, and consistency was assessed by presenting data in Bland–Altman plots. In these plots the difference between the WPM measurement and the SSM one is plotted against the mean value of the two measurements. Good agreement is indicated when the data points are scattered closely to the x-axis.

## Results

### Demographic and clinical data

Twelve volunteers (M: F = 3:9, 46 ± 14 yrs) were included in the studies. 9 of them were healthy volunteers and 3 were patient volunteers with poststernal discomfort or dysphagia, and finally confirmed as 1 achalasia and 2 ineffective esophageal motility (IEM) patients.

### Comparisons of manometric parameters between the two systems

**Figure 2:**
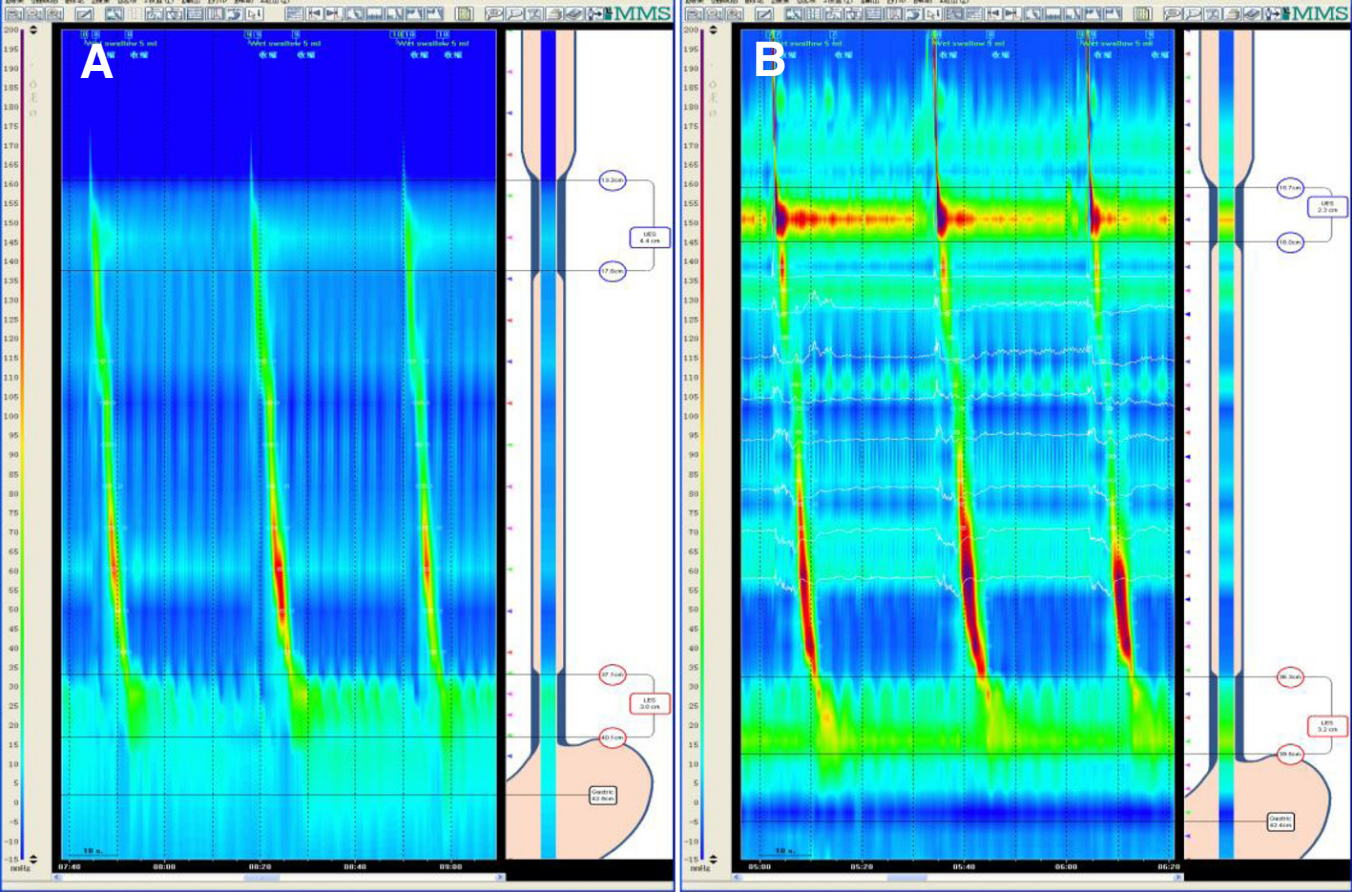
A: The Clouse Contour Plot of water-perfused manometry B: The Clouse Contour Plot of solid-state manometry.

**Figure 3:**
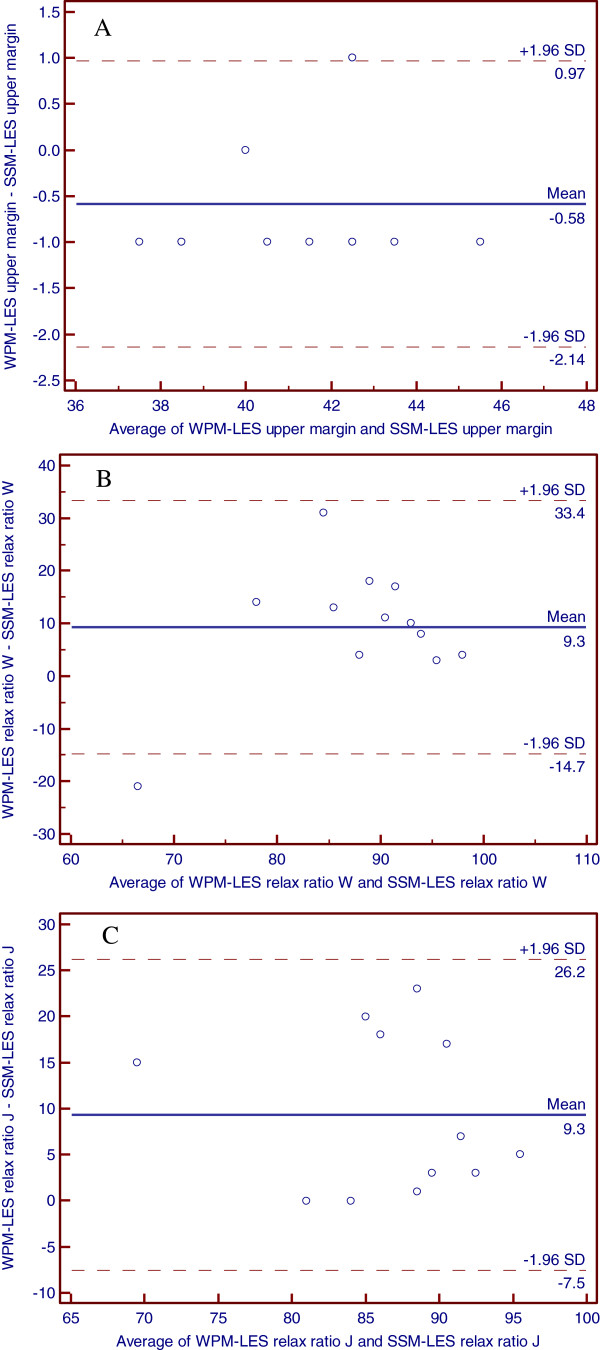
A: The Bland-Altman plots of LES upper margin between water-perfused manometry and solid-state manometry B: The Bland-Altman plots of LES relax ratio in wet swallow between water-perfused manometry and solid-state manometry C: The Bland-Altman plots of LES relax ratio in jelly pocket swallow between water-perfused manometry and solid-state manometry

Figure 2A- 2B showed the Clouse Contour Plot of WPM and SSM. During the wet swallows, there were significant differences between the two systems in the measurements of location of LES upper margin (Z = -2.11, P = 0.035) and LES relax ratio (Z = -2.20, P = 0.028). Table 1 showed the main motility measurements between groups during wet swallows. Further analysis showed the consistency in individual data of the two systems. Figure 3A- 3B presented Bland–Altman plots for location of LES upper margin and LES relax ratio. The data points are relatively closely scattered around the x-axis, indicating a small difference between the two measurements as compared to the mean of the two measurements.

**Table 1:** The differences of esophageal motility measurements between the 22-channel WPM and 36-sensor SSM during wet swallows (n = 12)

	**22-channel WPM**	**36-sensor SSM**	**Z value**	**P**
LES proximal margin (cm)	41.5(38-43)	42(39-43)	-2.11	0.035
LES length (cm)	4(4-4)	3.5(3-4)	0.00	1.000
LES rest pressure (mmHg)	13.5(10-21)	16.5(14-22.5)	-1.69	0.091
LES relax ratio (%)	97.5(92-98.5)	84(77-88.5)	-2.20	0.028
Distal esophageal body contraction intensity (mmHg)	68.5(40-85.25)	80(50-126.5)	-1.88	0.060
Distal esophageal body contraction duration (s)	3.85(2.9-4.2)	3.7(3.4-4.525)	-1.18	0.238
Proximal esophageal body contraction intensity (mmHg)	39(22-54)	51.5(40-62.75)	-1.96	0.050
Proximal esophageal body contraction duration (s)	3.05(2.6-3.275)	3.2(2.5-3.325)	-0.67	0.505
UES proximal margin (cm)	17(16-18)	16.5(16-17.25)	-0.81	0.417
UES length (cm)	4(4-5)	4(3-5)	-0.52	0.603
UES rest pressure (mmHg)	56.5(43-82.5)	77.5(60-113)	-1.88	0.060
UES relax ratio (%)	100(100-100)	100(97-100)	-0.76	0.446

Table 2 showed the main motility measurements during jelly pockets swallows. There was significant difference only in LES relax ratio measurement between the two systems (Z = -2.805, P = 0.005). However, Bland–Altman plots showed the good consistency in LES relax ratio of the two systems (Figure 3C).

**Table 2:** The differences of esophageal motility measurements between the 22-channel WPM and 36-sensor SSM during jelly pocket swallows (n = 12)

	**22-channel WPM**	**36-sensor SSM**	**Z**	**P**
LES relax ratio (%)	94.5(89-95.75)	83(77-88)	-2.805	0.005
Distal esophageal body contraction intensity (mmHg)	72(46-103.5)	100(70-144.25)	-1.883	0.060
Distal esophageal body contraction duration (s)	3.2(3-4.15)	3.9(3.3-5.4)	-1.785	0.074
Proximal esophageal body contraction intensity (mmHg)	51.5(38-57.75)	69.5(42-78)	-1.611	0.107
Proximal esophageal body contraction duration (s)	3.05(2.6-3.275)	3.2(2.5-3.325)	-0.432	0.665

**Figure 4:**
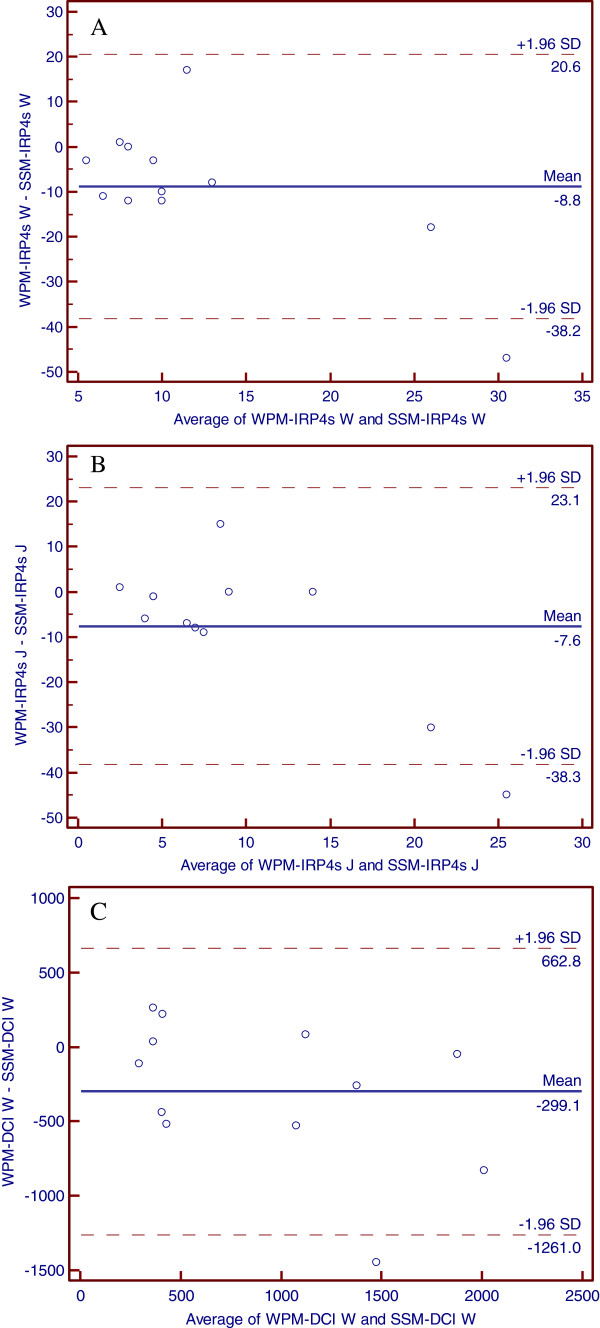
A: The Bland-Altman plots of IRP 4 s in wet swallow between water-perfused manometry and solid-state manometry B: The Bland-Altman plots of IRP 4 s in jelly pocket swallow between water-perfused manometry and solid-state manometry C: The Bland-Altman plots of DCI in wet swallow between water-perfused manometry and solid-state manometry.

When analyzing the topography parameters, significant difference presented in IRP4s (Z = -2.05, P = 0.041) during wet swallow (Table 3). There were difference, though no statistical significance, shown between the two systems measurements of IRP4s in jelly pocket swallow [3.5(3-6.75) vs. 9.5(5-12.5), Z = -1.788, P = 0.074] and DCI both in wet [636.5(344.8-1186) vs. 887.5(347.5-1607), Z = -1.84, P = 0.065] and jelly pocket swallows [817(587-1148) vs. 1305(797.3-1732), Z = -1.883, P = 0.060]. Further analysis indicated good agreement of the IRP4s, DCI measurements in the two systems (Figure 4A- 4C).

**Table 3:** The differences of topographic parameters’ measurements of Chicago Classification between the 22-channel WPM and 36-sensor SSM both during wet and jelly pocket swallows (n = 12)

	**22-channel WPM**	**36-sensor SSM**	**Z value**	**P**
CFV in wet swallow (cm/s)	7.25(6.325-9.25)	8.15(5.8-10.7)	-1.34	0.182
DCI in wet swallow (mmHg.s.cm)	636.5(344.8-1186)	887.5(347.5-1607)	-1.84	0.065
IRP in wet swallow 4 s (mmHg)	7.5(4-8.25)	13(7.75-16.25)	-2.05	0.041
CFV in jelly pocket swallow (cm/s)	5.45(4.775-6.025)	5.15(3.625-7.275)	-0.236	0.814
DCI in jelly pocket swallow (mmHg.s.cm)	817(587-1148)	1305(797.3-1732)	-1.883	0.060
IRP 4 s in jelly pocket swallow (mmHg)	3.5(3-6.75)	9.5(5-12.5)	-1.788	0.074

### Comparisons of patients’ tolerance and operators’ convenience

**Figure 5:**
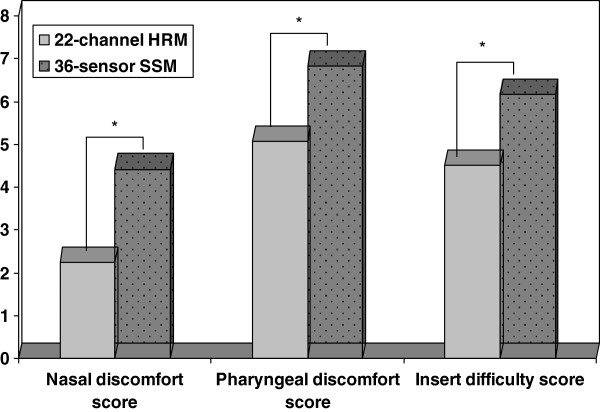
The VAS scores of patients’ nasal and pharngeal discomfort, as well as operators’ insert difficult indice were significantly higher when inserting solid-state catheter than that of water-perfused catheter.

The discomfort indices of subjects’ nasal sensation were higher when inserting the solid-state catheter [5(3.75-5)] than water-perfusion one [2.5(2-4)] (Z = -2.471, P = 0.013), as well as the discomfort indices of pharyngeal sensation [7.5(4.75-9) vs. 4.5(3.75-6.5)], (Z = -2.354, P = 0.019). The preparation time for WPC was 40(39-41) minutes, which was much longer than that for SSC (32.5(31.75-33)) minutes, (Z = -3.087, P = 0.002). And the nurses reported it’s much easier to insert WPC (Z = -3.126, P = 0.002). There were no significant differences of the motility process time between the two systems (Z = 0.000, P = 1.0). Figure 5 showed the differences of the scores of patients’ tolerance and operators’ convenience between the two systems.

## Discussion

In the current study, we compared the characteristics of the pressure measurements, patients’ discomfort indices in nose and pharynx, the preparation and operation time of the manometry between WPM and SSM with 36 sensors.

### Manometric parameters’ measurements in SSM clinically consisted well with that in WPM

The measurements showed the significant difference between the two systems in LES upper margin during wet swallow and the differences were confined in -1 to 1 cm as shown in Bland–Altman plots. This statistical difference of location of LES upper margin in the present study practically might play a limited role in clinical use. As it is widely known, the most important clinical significance of LES upper margin location is to help accurate placement of esophageal pH electrode for 24 hour pH monitoring. Measuring the exact location of the proximal border of the LES is presumably impossible because the LES is a ring that is localized in a diagonal plane in association with complex vector volumes [10] Therefore, a difference in distance of up to 2 cm may be determined by different radially-oriented openings during manometric measurement. The site of 3 cm above or below the exact location is commonly accepted in 24 h pH monitoring [11,12]. These findings indicate good clinical agreement of the measurements of LES upper margin location in the two systems.

The measurements of LES relax ratio and IRP4s also showed significant differences between the two systems. The Distal esophageal body contraction intensity and DCI presented difference as well, though without statistical significance. These differences might cause by the different pressure perception models of the two catheters. The measurement of SSC was taken from circumferential average pressure of the esophagus. However, the measurement taken with WPC came from single-side point, which mainly reflected unidirectional pressure of the esophagus. If the highest esophageal pressure was not exactly at the site of one of the WPC’s 1-cm readings, then the WPC may obtain a lower reading [3]. The second cause needed to be considered was the reproducibility of the parameters of HRM. Weak reproducibility might result in the significant differences in the two tests one after another. Boget et al. has reported the esophageal HRM yields reproducible results. Parameters that represent anatomic structures show better reproducibility than contraction wave parameters [13]. In our study, the differences did mostly exist in contraction wave parameters. However, the volunteers underwent the two manometries in random order and the bias may be reduced as much as possible. Pursnani et al. has demonstrated a high degree of correlation between measurements made with circumferential transducers compared to the sum of four unidirectional transducers in measuring resting LES pressures [6]. Our further analysis also presented the consistency of the measurements from the two systems by Bland–Altman plots. On the other side, these consistencies suggested the single-side point pressure could roughly reflect the circumferential average ones of LES relax ratio, as well as the other parameters (esophageal body contraction intensity, etc.), which displayed no significant statistical differences.

### Patients tolerated better with WPC and SSC presented more convenient

Patients considered more discomfort when insert the SSC, which is easy to understand and to be confirmed in the current study, because the external diameter of SSC with 36 sensors is larger than that of WPC made of PVC plastic. For the same reasons, the operators would feel more difficult to insert the SSC. The two systems need similar cleaning and sterilization times. Concerning the total preparation time, water-perfusion system needs to exhaust the air bubbles in the catheter, so it needed more time before the manometry process. The recent progress for the SSC is the single-use cover membrane with which the clean and sterilization process can be cancelled, however further increasing the cost of the manometry test by solid-state system.

There were some limitations in the current study. When regarding the quality of image, operators had a feeling that the SSM provided higher resolution image than that of WPM. It’s reasonable in theory because the SSC contains more closely spaced sensors than WPC. However, we have not yet discovered a method to quantify the differences. Additional image analysis techniques are needed for better understanding of this issue.

## Conclusions

In conclusions, most pressure measurements were consistent between WPM and SSM. Patients tolerated better with 22-channel WPC, while for operators, the 36-sensor SSC presented more convenient.

## Abbreviations

WPM: Water-perfusion manometry; SSM: Solid-state manometry; WPC: Water-perfusion catheter; SSC: Solid-state catheter; LES: Lower esophageal sphincter; UES: Upper esophageal sphincter; HRM: High resolution manometry; CFV: Contractile front velocity; DCI: Distal contractile integral; IRP: Integrated relaxation pressure.

## Competing interests

All the authors declare that they have no competing interests

## Authors’ contributions

LPD, Study concept and design; critical revision of the manuscript for content; KW, Acquisition of data; statistical analysis and interpretation of data; drafting of the manuscript; YG, Acquisition of data; ZWX, Contribution to design, Acquisition of data; ZJX, Acquisition of data. All authors read and approved the final manuscript.

## Pre-publication history

The pre-publication history for this paper can be accessed here:

http://www.biomedcentral.com/1471-230X/12/157/prepub
